# Should the ultrasound probe replace your stethoscope? A SICS-I sub-study comparing lung ultrasound and pulmonary auscultation in the critically ill

**DOI:** 10.1186/s13054-019-2719-8

**Published:** 2020-01-13

**Authors:** Eline G. M. Cox, Geert Koster, Aidan Baron, Thomas Kaufmann, Ruben J. Eck, T. Corien Veenstra, Bart Hiemstra, Adrian Wong, Thomas C. Kwee, Jaap E. Tulleken, Frederik Keus, Renske Wiersema, Iwan C. C. van der Horst, SICS Study Group, Geert Koster, Frederik Keus, Iwan C. C. van der Horst, Willem Dieperink, Roos Bleijendaal, Yasmin F. Cawale, Ramon P. Clement, Devon Dijkhuizen, Ruben J. Eck, Bart Hiemstra, Anja Haker, Casper D. H. Hilbink, Thomas Kaufmann, Martiene Klasen, Manon Klaver, Laura J. Schokking, Victor W. Sikkens, Madelon Vos, Justin Woerlee, Renske Wiersema

**Affiliations:** 1 Department of Critical Care, University Medical Center Groningen, University of Groningen, P.O. Box 30.001, 9700 RB Groningen, The Netherlands; 2 Emergency, Cardiovascular, and Critical Care Research Group, Centre for Health and Social Care Research, Kingston University and St George’s University, London, UK; 3 Department of Anesthesiology, University Medical Center Groningen, University of Groningen, Groningen, The Netherlands; 4 Department of Internal Medicine, University Medical Center Groningen, University of Groningen, Groningen, The Netherlands; 5 Department of Anaesthesiology and Intensive Care, Royal Surrey County Hospital, Guildford, UK; 6 Department of Radiology, University Medical Center Groningen, University of Groningen, Groningen, The Netherlands; 7 Department of Intensive Care, Maastricht University Medical Center+, Maastricht University, Maastricht, The Netherlands

**Keywords:** Prospective study, Lung ultrasound, Auscultation, Pulmonary edema, Clinical examination, Critical care, Diagnostic accuracy

## Abstract

**Background:**

In critically ill patients, auscultation might be challenging as dorsal lung fields are difficult to reach in supine-positioned patients, and the environment is often noisy. In recent years, clinicians have started to consider lung ultrasound as a useful diagnostic tool for a variety of pulmonary pathologies, including pulmonary edema. The aim of this study was to compare lung ultrasound and pulmonary auscultation for detecting pulmonary edema in critically ill patients.

**Methods:**

This study was a planned sub-study of the Simple Intensive Care Studies-I, a single-center, prospective observational study. All acutely admitted patients who were 18 years and older with an expected ICU stay of at least 24 h were eligible for inclusion. All patients underwent clinical examination combined with lung ultrasound, conducted by researchers not involved in patient care. Clinical examination included auscultation of the bilateral regions for crepitations and rhonchi. Lung ultrasound was conducted according to the Bedside Lung Ultrasound in Emergency protocol. Pulmonary edema was defined as three or more B lines in at least two (bilateral) scan sites. An agreement was described by using the Cohen *κ* coefficient, sensitivity, specificity, negative predictive value, positive predictive value, and overall accuracy. Subgroup analysis were performed in patients who were not mechanically ventilated.

**Results:**

The Simple Intensive Care Studies-I cohort included 1075 patients, of whom 926 (86%) were eligible for inclusion in this analysis. Three hundred seven of the 926 patients (33%) fulfilled the criteria for pulmonary edema on lung ultrasound. In 156 (51%) of these patients, auscultation was normal. A total of 302 patients (32%) had audible crepitations or rhonchi upon auscultation. From 130 patients with crepitations, 86 patients (66%) had pulmonary edema on lung ultrasound, and from 209 patients with rhonchi, 96 patients (46%) had pulmonary edema on lung ultrasound. The agreement between auscultation findings and lung ultrasound diagnosis was poor (*κ* statistic 0.25). Subgroup analysis showed that the diagnostic accuracy of auscultation was better in non-ventilated than in ventilated patients.

**Conclusion:**

The agreement between lung ultrasound and auscultation is poor.

**Trial registration:**

NCT02912624. Registered on September 23, 2016.

## Introduction

Physicians are trained to use auscultation as part of clinical examination in routine care for critically ill patients. Auscultation is accepted as one of the essential components of the clinical examination. Frequent pathologies encountered in the critically ill are pulmonary edema and pneumonia; both present with an increase in alveolar fluid and often coexist. Crepitations and rhonchi can be present in patients with pulmonary edema [1]. In recent years, clinicians have started to consider lung ultrasound (LUS) as a useful diagnostic tool for a variety of pulmonary pathologies [2–4]. An increasing body of evidence supports the use of LUS in diagnosing pulmonary edema and/or pneumonia [5]. Several studies have shown the diagnostic value of LUS in patients with dyspnea or specific diagnoses, such as pneumothorax, high-altitude pulmonary edema, and cardiogenic pulmonary edema [6–10]. LUS has even been suggested to be superior to chest radiography (X-ray) and comparable to chest computed tomography (CT) scan for the diagnosis of pulmonary edema and increased alveolar fluid (commonly referred to as interstitial syndrome) [3, 8]. However, few studies have compared LUS to pulmonary auscultation, even while the stethoscope still constitutes the majority of contemporary practice [11–13].

In critically ill patients, auscultation might be challenging as dorsal lung fields are difficult to reach in supine-positioned patients, and the environment is often noisy. No studies have prospectively compared auscultation with LUS in the intensive care unit (ICU) setting. Accordingly, the aim was to compare the agreement of LUS with pulmonary auscultation for the detection of pulmonary edema in acutely admitted ICU patients. We hypothesized that auscultation for pulmonary edema would have insufficient agreement compared to LUS.

## Methods

### Design and setting

This was a planned sub-study of the Simple Intensive Care Studies-I (SICS-I), a single-center, prospective observational study designed to evaluate the diagnostic and prognostic value of combinations of clinical examination and critical care ultrasound (CCUS), in critically ill patients [14]. This sub-study and a prespecified hypothesis were added to the SICS-I study [14]. The local institutional review board (Medisch Ethische Toetsingscommissie of the University Medical Center Groningen (UMCG)) approved the study (M15.168207). This manuscript was reported according to the Standards for Reporting of Diagnostic Accuracy Studies guidelines [15].

### Participants

All acutely admitted patients who were 18 years and older with an expected ICU stay of at least 24 h were eligible for inclusion. Patients were excluded if their ICU admission was planned; if acquiring research data interfered with clinical care due to, for example., continuous resuscitation efforts (e.g., mechanical circulatory support); or if consent was not obtained. In this sub-study, we selected a convenience sample of patients who had bilateral LUS images in at least two scan sites.

### Variables

All included patients underwent clinical examination followed by CCUS within the first 24 h of their ICU admission. The researchers were senior medical students and junior residents trained by cardiologist-intensivists for both clinical examination and CCUS before contributing to the study. Training included self-study of theory on how to perform auscultation and lung ultrasound, at least 2 h hands-on training from cardiologists-intensivists, practice on healthy individuals during practical sessions, and supervised clinical examination and CCUS in the first 20 patients.

Data from the clinical examination was prospectively collected based on definitions in the protocol, including the presence of crepitations and rhonchi [14]. Abnormal auscultation was defined as the presence of crepitations and/or rhonchi at any of the sites. Pulmonary edema was defined as the presence of three or more B lines; diffuse pulmonary edema was defined as edema in two or more scan sites of LUS bilaterally [16].

**Fig. 1:**
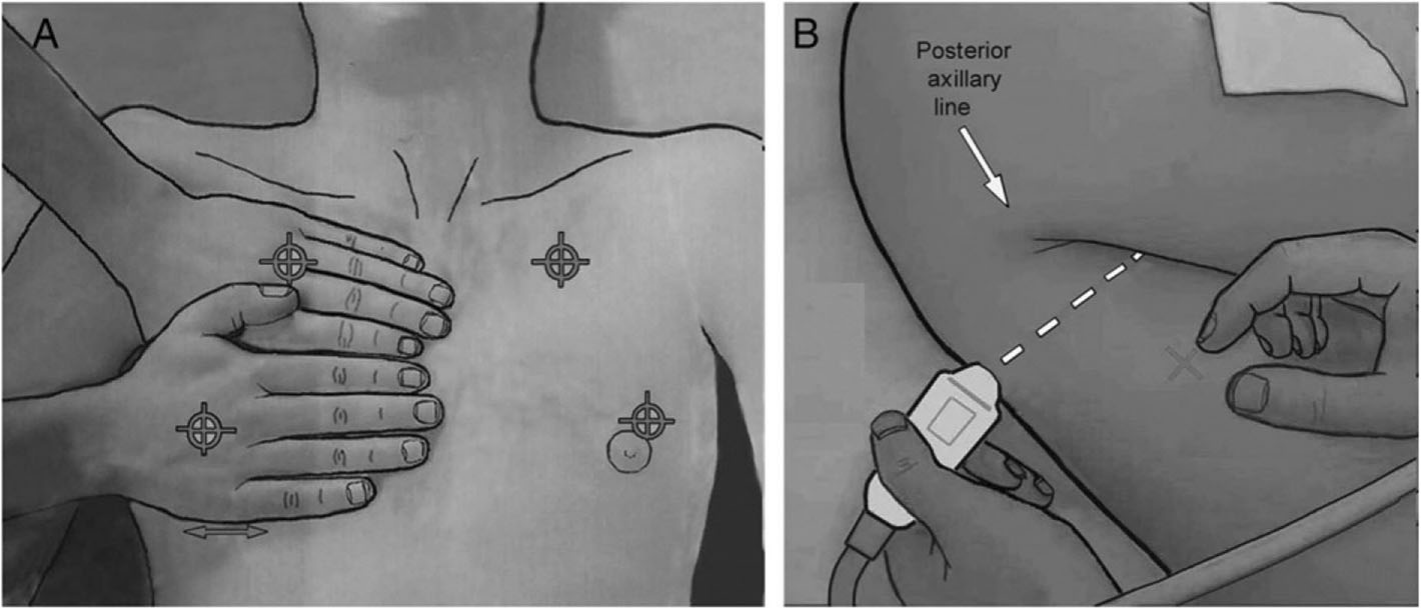
The six scan sites according to the BLUE-protocol [18]

Auscultation was performed of the anterior and axillary lung fields in each hemithorax with the patient in a supine position. Subsequently, CCUS was performed following a predefined protocol using a phased array probe (M3S or M4S) set at a frequency of 3.6 MHz, a depth of 15 cm, and maximal image width (Vivid-S6, GE Healthcare, London, UK) [17]. LUS was performed using the Bedside Lung Ultrasound in Emergency (BLUE) protocol, assessing six scan sites per patient (superior, inferior, and lateral, bilateral) (Fig. 1). In each scan site, the numbers of B lines (0–5) were recorded [18]. Measurements were subsequently conducted by researchers, who were not involved in patient care. Researchers were instructed not to share their findings with the attending physicians, so that these were used for research purposes only.

### Statistical analyses

The overall statistical methods were described in the predefined statistical analysis plan (SAP) of the main study (NCT02912624). Continuous variables were reported as means with standard deviation (SD) or median with interquartile range (IQR) depending on the distributions. Categorical data were presented in proportions. Student’s *t* test, Mann-Whitney *U* test, or the chi-square tests were used as appropriate. The agreement between LUS and auscultation for pulmonary edema was described by using the Cohen *κ* coefficient. Sensitivity, specificity, positive predictive value (PPV), negative predictive value (NPV), and diagnostic accuracy of lung ultrasound against auscultation to detect pulmonary edema were calculated. Analyses were performed using Stata version 15 (StataCorp, College Station, TX, USA). A subgroup analysis was performed to assess whether these results were robust in patients who were not mechanically ventilated. We performed a sensitivity analysis to assess the agreement and diagnostic accuracy of LUS for pulmonary edema on chest X-ray, in patients where a chest X-ray was available shortly before or after study inclusion (i.e., on the same day).

The SICS-I was designed to address multiple hypotheses on six different outcomes, and therefore, the pulmonary edema outcome was adjusted for multiple hypothesis testing. We refer to our SAP for more details, but in short, a *p* value of 0.015 indicated statistical significance and *p* values between 0.015 and 0.05 indicated suggestive significance with an increased family-wise error rate [19]. For secondary or sensitivity analyses, a *p* value below 0.05 indicated statistical significance due to the hypothesis-generating purpose. Accordingly, the primary analyses are presented with 98.5%CIs and secondary (subgroup) analyses with 95%CIs.

## Results

**Fig. 2:**
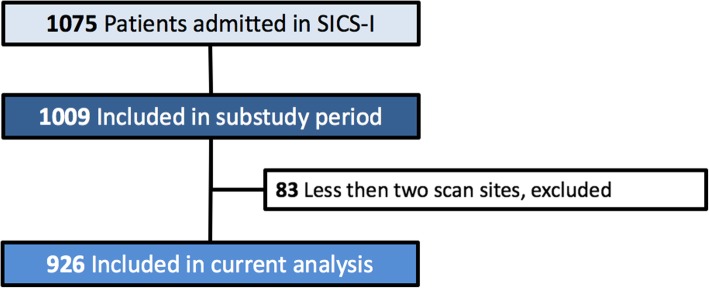
Flowchart. Less than two scan sites meaning if less than two out of six scan sites or no bilateral scan sites of LUS were available, the presence of pulmonary edema could not be assessed

This SICS-I sub-study started on September 15, 2015, and continued until July 22, 2017, during which 1009 patients were included. A total of 149 patients (15%) were excluded because no bilateral or less than 2 scan sites were scanned due to emphysema, drains, or wound dressings hampering the ultrasound windows, leaving 926 patients (85%) for the analysis (Fig. 2). Baseline characteristics of all patients are shown in Table 1.

**Table 1:** Baseline characteristics of all included patients

	*N* = 926
Age, years (SD)	62 (14)
Gender, male (%)	598 (64)
Height, cm (SD)	176 (10)
Weight, kg (SD)	83 (18)
Mechanical ventilation, *n* (%)	537 (57)
Vasoactive medication, *n* (%)	461 (49)
APACHE IV score, mean (SD)	76 (29)
Admission type	
- Surgical, *n* (%)	292 (31)
- Medical, *n* (%)	645 (69)
Outcomes	
- Length of stay, days	3.3 (1.9–6.8)
- 90-day mortality, n (%)	249 (27)

### Findings of lung ultrasound and auscultation

The criteria for pulmonary edema diagnosed by LUS were met in 307 of 926 patients (33%). In 156 of these patients (51%), auscultation was normal. A total of 302 of 926 patients (32%) had pulmonary edema diagnosed by pulmonary auscultation. From these patients, 151 patients (50%) had pulmonary edema on LUS. Of the 302 patients with pulmonary edema on auscultation, 130 patients had crepitations and 209 patients had rhonchi.

From 130 patients with crepitations, 86 patients (66%) had pulmonary edema on LUS, and of the 209 patients with rhonchi, 96 patients (46%) had pulmonary edema on LUS. The agreement between auscultation and LUS was poor (*κ* statistic 0.25).

### Diagnostic performance

Diagnostic performance measures of crepitations, rhonchi, and auscultation for the detection of pulmonary edema are displayed in Table 2. The sensitivity of crepitations was 66% (98.5% CI 55–76), specificity was 71% (98.5% CI 67–75), positive predictive value was 28% (98.5% CI 22–34), and negative predictive value was 93% (98.5% CI 90–95). The overall diagnostic accuracy of crepitations was 72% (98.5% CI 69–74). The sensitivity of rhonchi was 47% (98.5% CI 39–56), specificity was 69% (98.5% CI 65–74), positive predictive value was 31% (98.5% CI 25–38), and the negative predictive value was 82% (98.5% CI 77–85). The overall diagnostic accuracy of rhonchi was 64% (98.5% CI 61–67).

**Table 2:** Test characteristics of specific findings compared to LUS in all patients

	Abnormal, *N*	Total, *N*	Diagnostic performance in % (98.5% confidence intervals)				
			Sensitivity	Specificity	PPV	NPV	Diagnostic accuracy
Crepitations	130	917	66 (55–76)	71 (67–75)	28 (22–34)	93 (90–95)	72 (69–74)
Rhonchi	209	913	47 (39–56)	69 (65–74)	31 (25–38)	82 (77–85)	64 (61–67)
Auscultation	302	926	52 (45–59)	74 (70–79)	49 (42–56)	76 (72–80)	67 (64–70)

Abnormal auscultation was defined as the presence of crepitations and/or rhonchi at any of the sites

The sensitivity of abnormal auscultation overall was 52% (98.5% CI 45–59), specificity was 74% (98.5% CI 70–79), positive predictive value was 49% (98.5% CI 42–56), and the negative predictive value was 76% (98.5% CI 72–80). The overall diagnostic accuracy of auscultation was 67% (98.5% CI 64–70).

### Sensitivity analysis

Diagnostic accuracy of auscultation improved if patients were not mechanically ventilated (Table 3). The overall accuracy for auscultation was 69% (95% CI 64–74) in non-mechanically ventilated patients and 67% (98.5%CI 64–70) in all patients (*p* < 0.001). The overall accuracy for crepitations was 71% (95% CI 67–76) for rhonchi and 66% (95%CI 61–71) in non-ventilated patients. The agreement between auscultation and LUS improved in non-mechanically ventilated patients (*κ* statistic 0.31).

**Table 3:** Test characteristics of specific findings compared to LUS in non-mechanically ventilated patients

	Abnormal, *N*	Total, *N*	Diagnostic performance in % (95% confidence intervals)				
			Sensitivity	Specificity	PPV	NPV	Diagnostic accuracy
Crepitations	73	387	36 (28–45)	90 (85–94)	66 (55–75)	73 (70–75)	71 (67–76)
Rhonchi	70	384	28 (21–36)	87 (82–91)	54 (44–64)	69 (66–71)	66 (61–71)
Auscultation	124	391	51 (43–60)	79 (73–84)	56 (49–63)	75 (72–79)	69 (64–74)

Abnormal auscultation was defined as the presence of crepitations and/or rhonchi at any of the sites

Radiologists’ reports assessing the chest X-ray were analyzed in a subset of 315 patients as this was part of the standard ICU management until November 21, 2016. The baseline characteristics of these patients were comparable to the overall population (Additional file 1: Table S1). The median time lag between LUS and chest X-ray was 4 h (2–7 h). In 89 of these patients (28%), the radiologist reported the diagnosis of edema; in 6 patients (2%), it was unclear; and in 220 patients (70%), there was no pulmonary edema on chest X-ray according to the radiologist (Additional file 1: Table S2). The agreement and diagnostic accuracy of LUS for pulmonary edema as diagnosed on chest X-ray were limited (*κ* statistic 0.12; Additional file 1: Table S3).

## Discussion

In this prospective observational study, we found poor agreement between auscultation and LUS for the diagnosis of pulmonary edema in acutely admitted critically ill patients.

Several previous studies focused on the diagnostic accuracy of LUS compared to other imaging modalities, such as chest X-ray and CT scan [4, 10, 20]. However, few studies have compared the diagnostic accuracy of LUS with the stethoscope, one of the most frequently used instruments at the bedside. Lichtenstein et al. prospectively compared the diagnostic performance of auscultation, LUS, and chest X-ray for detecting alveolar consolidation and alveolar-pulmonary edema with CT scan in 32 patients with acute respiratory distress syndrome and in 10 healthy volunteers [13]. The authors found that auscultation had a diagnostic accuracy of 55% for alveolar-pulmonary edema, which corresponds fairly to the 67% accuracy in our study [13]. In that study, LUS had a diagnostic accuracy of 97% for alveolar consolidation and 95% for alveolar-pulmonary edemas, and chest X-ray had a diagnostic accuracy of 75% for alveolar consolidation and 72% for alveolar-pulmonary edema [13]. In a sensitivity analysis, we observed that the agreement and diagnostic accuracy of LUS for pulmonary edema were limited when compared to chest X-ray, which is in line with other studies [1].

Another study by Torino et al. prospectively investigated the agreement between auscultation and LUS in non-admitted patients before and after undergoing hemodialysis [11]. The authors similarly found a very poor agreement (*κ* statistic 0.16, in this study *κ* statistic 0.25) between the presence of crepitations on auscultation and the presence of B lines on LUS in a total of 1106 measurements in 79 patients [11]. Although their population seems different to ours, patients receiving dialysis may also suffer from pulmonary edema as a consequence of fluid overload. Their results and conclusions are similar to ours, and therefore, these observations may be generalizable to populations beyond the critically ill.

We found that the diagnostic accuracy of auscultation improved if patients were not mechanically ventilated; no previous study has reported this finding. Acoustic disturbances caused by the ventilators might explain the complicated appreciation of subtle auscultation findings.

### Implications and generalizability

Improved diagnostic accuracy for detecting pulmonary edema could lead to improved treatment leading to increased benefits and decreased harms for the patient. In critically ill patients, typically multiple pathophysiological processes are co-occurring at the same time, which hampers the extrapolation of the test characteristics for diagnosing abnormalities in these patients, such as pulmonary edema. As some physicians still use auscultation to detect pulmonary edema, we think our study clarifies that auscultation may not be as reliable for detecting pulmonary edema as classically perceived, especially in the ICU. Ultrasonography becomes increasingly available, and our data add nuance to the discussion surrounding how this technology might be properly integrated into clinical practice in the care of the critically ill. These observations encourage further research of LUS; the need for external validation remains to increase the generalizability of this diagnostic modality.

### Limitations

Several limitations of this study must be acknowledged. First, the clinical examination and ultrasonography were conducted as early as possible after ICU admission which limits the applicability of use in patients with prolonged admission. Further studies should explicate how auscultation and LUS compare in other departments and more specifically other pathologies such as a pneumothorax. Second, we were not able to validate all our LUS assessments by experts, also because there are no reference standards for the interpretation of LUS. Chest X-ray and CT are other diagnostic methods that are frequently used for the assessment of pulmonary edema. However, previous studies have suggested that LUS is superior to chest X-ray and comparable to chest CT scan for diagnosing pulmonary edema [3, 8]. Therefore, we decided not to use these modalities as a reference standard and only included a sensitivity analysis of chest X-ray. We limited LUS reporting to the number of B lines per field and did not use further qualitative commentary. Third, the auscultation was not standardized. During clinical examination, researchers performed both auscultation and LUS; however, in contrast to LUS, we did not describe in detail the location of auscultation. In practice, these were similar to the LUS scan sites. Therefore, we think the influence on our results is minimal. Also, the researchers only specified whether they heard significant crepitation or rhonchi on auscultation. Other abnormal breathing sounds were not recorded and we only documented their overall presence or absence; we are unable to compare auscultation with LUS for each specific scan site. In addition, ideally, we ask the patient to cough to distinguish between rhonchi and/or crepitations. Unfortunately, the large majority of the patients in the ICU are not cooperative with this request. Fourth, even though the researchers who performed the measurements were not involved in patient care, they were not blinded for patient information, such as admission diagnoses, other clinical variables and the results of auscultation when performing the CCUS. However, as ultrasonography was always performed after auscultation, we believe it is proper to discuss this potential source of bias but do not believe that it substantially influenced our results due to the objective nature of B line appearance. Fifth, since researchers were senior medical students and junior residents, auscultation by more experienced medical doctors could potentially improve the diagnostic accuracy. Last, 83 (8%) patients were excluded from the analyses due to the absence of LUS or auscultation data. However, the relatively small proportion of this excluded patient group makes it unlikely that excluded patients would have altered the conclusions. Despite the potential biases and limitations, we showed that the agreement between auscultation and lung ultrasound was poor. This is important as current data is scarce on the diagnostic value of new non-invasive bed tools such as CCUS, especially in comparison with clinical examination in critically ill patients.

## Conclusions

The agreement between auscultation and LUS for detecting pulmonary edema is poor. As some physicians still use auscultation to detect pulmonary edema, this study clarifies that auscultation may not be as reliable for detecting pulmonary edema as classically perceived, especially in the ICU.

## Supplementary information

**Additional file 1: Table S1.** Baseline characteristics of patients with and without chest X-ray. **Table S2.** Pulmonary edema as diagnosed on chest-X ray and LUS. * We have excluded 6 patients with a chest X-ray due to unclear images. **Table S3.** Diagnostic performance of LUS for pulmonary edema on chest X-ray.

## Data Availability

The datasets used and/or analyzed during the current study are available from the corresponding author on reasonable request.

## Abbrevations

APACHE IV: Acute Physiology and Chronic Health Evaluation
BLUE-protocol: Bedside Lung Ultrasound in Emergency protocol
CCUS: Critical care ultrasound
CT: Computerized tomography
ICU: Intensive care unit
IQR: Interquartile range
LUS: Lung ultrasound
NPV: Negative predictive value
PPV: Positive predictive value
SAP: Statistical analysis plan
SD: Standard deviation
SICS: Simple Intensive Care Studies
X-ray: Radiography
