# Supplementary material for: Should the ultrasound probe replace your stethoscope? A SICS-I sub-study comparing lung ultrasound and pulmonary auscultation in the critically ill
Source: Crit Care. 2020 Jan 13;24:14. doi: 10.1186/s13054-019-2719-8 (PMC6958607; doi:10.1186/s13054-019-2719-8)
Supplement: Supplementary file 1 — Additional file 1: Table S1. Baseline characteristics of patients with and without chest X-ray. Table S2. Pulmonary edema as diagnosed on chest-X ray and LUS. * We have excluded 6 patients with a chest X-ray due to unclear images. Table S3. Diagnostic performance of LUS for pulmonary edema on chest X-ray. [file 13054_2019_2719_MOESM1_ESM.docx]

**Additional file 1**

**Table S1.** Baseline characteristics of patients with and without chest X-ray

| Variable | All patients  *n = 926* | Patients with  chest x-ray  *n = 315* |
| --- | --- | --- |
| Age, years (SD) | 62 (14) | 61 (15) |
| Gender, male (%) | 598 (64) | 216 (69) |
| Height, cm (SD) | 176 (10) | 177 (10) |
| Weight, kg (SD) | 83 (18) | 84 (18) |
| Mechanical ventilation, n (%) | 537 (57) | 207 (65.7) |
| Vasoactive medication, n (%) | 461 (49) | 173 (55) |
| APACHE IV - score, mean (SD) | 76 (29) | 79 (32) |
| Admission type   - Surgical, n (%) - Medical, n (%) | 292 (31)  645 (69) | 88 (28)  227 (72) |
| Outcomes   - Length of stay, days - 90-day mortality, n (%) | 3.3 (1.9-6.8)  249 (27) | 3.6 (2.1 -7.3)  87 (28) |

**Table S2.** Pulmonary edema as diagnosed on chest-X ray and LUS

| Pulmonary edema on chest X-ray | Pulmonary edema on LUS | | |
| --- | --- | --- | --- |
|  | **Positive** | **Negative** | **Total** |
| Positive | 49 | 40 | 89 |
| Negative | 88 | 132 | 220 |
| Total | 137 | 172 | 309* |

* We have excluded 6 patients with a chest X-ray due to unclear images

**Table S3.** Diagnostic performance of LUS for pulmonary edema on chest X-ray

|  | **Abnormal** | **Total** | **Diagnostic performance in % (95% confidence intervals)** | | | | |
| --- | --- | --- | --- | --- | --- | --- | --- |
|  | **N** | **N** | **Sensitivity** | **Specificity** | **PPV** | **NPV** | **Diagnostic accuracy** |
| **Pulmonary edema** | 89 | 309 | 55 (44-66) | 60 (53-67) | 36 (30-42) | 77 (72-81) | 59 (53-64) |
